# Variability and Performance Study of the Sound Absorption of Used Cigarette Butts

**DOI:** 10.3390/ma12162584

**Published:** 2019-08-14

**Authors:** Valentín Gómez Escobar, Guillermo Rey Gozalo, Carlos J. Pérez

**Affiliations:** 1 Departamento de Física Aplicada, Escuela Politécnica, Universidad de Extremadura, Avda. de la Universidad s/n, 10003 Cáceres, Spain; 2 Facultad de Ciencias de la Salud, Universidad Autónoma de Chile, 5 Poniente 1670, 3460000 Talca, Región del Maule, Chile; 3 Departamento de Matemáticas, Facultad de Veterinaria, Universidad de Extremadura, Avda. de la Universidad s/n, 10003 Cáceres, Spain

**Keywords:** sound absorber, cigarette butts, sustainable material, recycling, variability analysis

## Abstract

There has been increasing interest in new sustainable materials that can be used as construction materials. Among them, sound-absorbing materials have an important role in both acoustical room conditioning and in room insulation. As a proposal for recycling, one of the most common residues in the world, cigarette butts, is studied. Samples were prepared with used cigarette butts as acoustical absorbent materials. Several samples were prepared and grouped by similarity. Variability analyses of the samples prepared in each group were performed. Moreover, the analysis of some possible influences on absorption properties, such as the length of butts, presence of burnt regions, presence of wrapping paper, etc., were analyzed. The results show the potentiality of this residue to be used as an acoustical absorbent since the absorption coefficients found are greater than 0.8 for frequencies over 2000 Hz. The observed variability in the study group and samples can be considered low, as it was below 2% for the major part of frequencies. Influences on the absorption coefficient, for both the length and status of the butts, were statistically confirmed.

## 1. Introduction

Among the different construction materials, there has been special interest in sound absorption materials [1] that can help both in adapting room acoustics to a particular use and in achieving better insulation characteristics of different building partitions. The use of industrially manufactured mineral wools is widely extended for sound absorption in construction although, in recent years, some alternative materials have been proposed for acoustical absorption [2]. Thus, there are studies on the acoustical behavior of indoor or outdoor plants [3,4,5], materials made of natural fibers [6,7,8,9,10], sheep wool [11], cork wastes [12], and olive pruning wastes [13], to name but a few.

In recent decades, there have been increasing efforts to recycle all the waste materials produced by human activities. This recycling practice has also emerged in construction materials. Thus, as a more sustainable option, among the different proposed alternative materials for sound absorption, some are based on recycling materials [12,13,14,15]. These recycled materials have the additional value of contributing to the elimination of waste materials produced by human activities.

One of the major components of human debris is used cigarette butts. Cigarette filters were introduced (in the middle of the past 20th century) to reduce the incidence of diseases in smokers. However, this had a collateral effect, i.e., the fact that the cellulose diacetate (substance from which filters are mainly composed) is not biodegradable and has only partial photodegradation makes used cigarette butts an important environmental concern. It must be considered that, throughout the world, billons of cigarettes are consumed every year [16,17], and it is expected that the consumption will increase by more than 50% by 2025 due to the increase in world population and tobacco production [18]. The problem due to this residue is enlarged due to the unfortunately common habit of smokers to throw cigarette butts on the ground [19,20]. Thus, in studies on the debris composition of places of garbage collection, such as bins, containers, etc., but also in studies of different human environments (streets, beaches, etc.), butts are commonly the main component in number and even sometimes in weight [21,22,23]. The butts thrown on the ground are transported by rain or river water to coastal areas, increasing their environmental impact. This environmental impact of used cigarette butts is related both to their chemical composition [24] and to the difficult degradation of cellulose diacetate, as mentioned. Some of the 130 chemicals found in cigarette butts have been described. In addition, many more chemicals (40,000–100,000) have been identified in cigarette smoke, some of which could be retained in cigarette butts [25]. These chemicals can easily leach into water, making these waters toxic for some organisms [24,26,27,28]. Consequently, cigarette butts are a concern in terms of the environment and public health.

There are some recycling proposals concerning cigarette butts, such as using them as part of the composition of bricks [29] or supercapacitors [30], or using their washing waters as chemical inhibitors [31] or as insecticide [32], etc. A revision of different proposals for recycling used butts has recently been carried out [33]. However, these proposals would not be enough to recycle all the annual production of this major residue and, certainly, new proposals are welcome. In this sense, our research group is working on the applying used cigarette butts as acoustical absorbers.

Some preliminary studies have shown the potential of used cigarettes butts for the preparation of acoustic absorbers [34,35]. However, additional studies are needed to analyze the application of used cigarette butts for this purpose. For example, whether the variability of the status (used and/or burned), diameter, and length of the butts can influence their acoustic properties. Additionally, the effect of the paper covering the cigarette filters is a factor to consider. Moreover, the comparison between used and unsmoked butts can also be interesting. These considerations are dealt with in this paper. 

Taking into account the abovementioned information, the influence of the status, length, and paper wrapping on the spectrum of sound absorption are analyzed in the present study. For this purpose, preliminary and careful determination of the characteristics of the cigarette butts was carried out. Moreover, an appropriate number of similar samples were prepared, and the analysis of the different measured absorption coefficients is enriched through statistical analysis. A relevant influence of these factors on the absorption properties of the samples prepared with butts would imply allow for selective collection and planning for these residues.

## 2. Materials and Methods 

### 2.1. Instrumentation for Acoustic Absorption Determination

The determination of the sound absorption coefficient of different samples was carried out using an impedance tube, following the two-microphone transfer function method described in the ISO 10534-2 standard [36]. 

**Figure 1:**
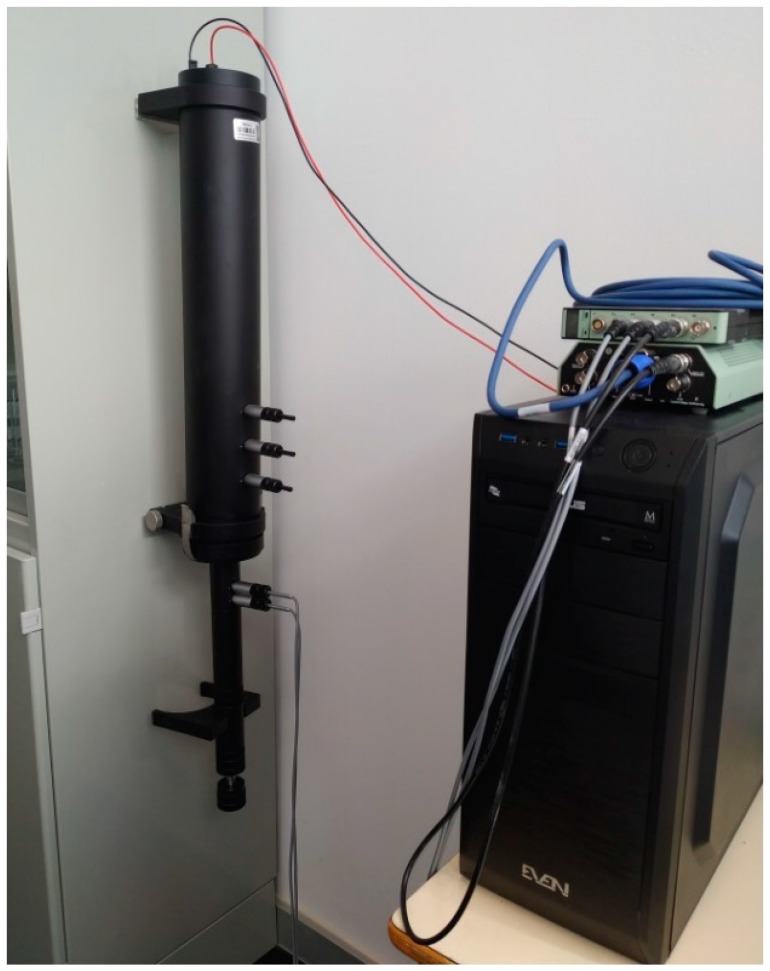
Impedance tube: experimental setup.

The measurements were conducted using an Impedance Tube Kit (Type 4206, Brüel & Kjaer, Nærum, Denmark), equipped with two one-quarter-inch condenser microphones (Type 4187). As the prepared samples could be considered non-consolidated, the tube was placed in a vertical position (see Figure 1). The signals were analyzed using a portable PULSE System (Brüel & Kjaer, Nærum, Denmark) with four input data channels (Type 3560-C). A sample holder, with a diameter of 29 mm, was used (with a validity in the frequency range of 500 Hz to 6.4 kHz).

### 2.2. Preparation of Samples

Smoked cigarette butts were collected from ashtrays or the ground, and the remaining non-smoked tobacco was then manually separated. Only the cigarette butts were taken. 

These remaining butts were nonhomogeneous. The length, diameter, presence of burnt regions, compression exerted by user’s fingers, humidity, etc., could be different in each butt. In order to avoid part of the variability introduced by these characteristics, for this study, the cigarette butts were separated into equivalent groups prior to the preparation of the samples. The diameter and length of all the cigarette butts were measured before sample preparation. While there are some brands of cigarettes that use a narrower diameter, for the present study, a diameter of only around 7.5–8.0 mm was used, as it was the most common diameter among the available cigarette butts.

As the diameter of the butts can be considered very similar, the main variables to consider were the initial status of the butts (used, or unsmoked, and/or burnt) and their length. Three length groups were considered:

- Length 1 (L1): length = 15.13 ± 0.40 mm; diameter = 7.95 ± 0.07 mm.
- Length 2 (L2): length = 20.67 ± 0.22 mm; diameter = 7.64 ± 0.06 mm.
- Length 3 (L3): length = 26.61 ± 0.13 mm; diameter = 7.59 ± 0.04 mm.

The following four statuses were established:

- Condition 1 (C1): Smoked cigarette butts without burnt regions. Burnt regions were considered when some hard structure was formed in the cigarette butts due to the burning of a part of the filter.
- Condition 2 (C2): Smoked cigarette butts with burnt regions.
- Condition 3 (C3): Smoked cigarette butts without burnt regions and without the paper that wrapped the filter. The wrapping paper was removed carefully, avoiding loss of the fibrous material of the filter.
- Condition 4 (C4): Unsmoked cigarette butts.

Each sample was prepared by manually placing 10 cigarette butts in the sample holder of the impedance tube. This number of butts was selected to avoid high compression of butts in the samples. At least 10 cigarette butts samples from each group were prepared in order to have an adequate number of samples to allow for subsequent statistical analyses. The different groups of samples measured are presented in Table 1, indicating the number of samples prepared (each one with at least 10 different samples, as mentioned), density of samples, and length and diameters of the used cigarette for each group. Porosity of samples was determined considering the porosity of some individual butts (calculated by a helium pycnometer) and considering a spherical geometry for the cigarette butts of samples.

**Figure 2:**
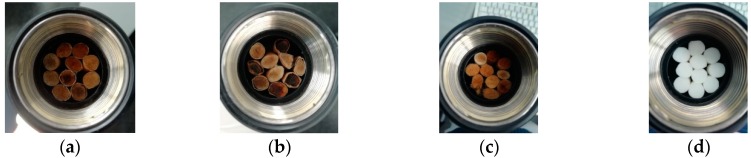
Example pictures of some of the prepared samples: (**a**) Sample from C1 cigarette butts; (**b**) Sample from C2 cigarette butts; (**c**) Sample from C3 cigarette butts; (**d**) Sample from C4 cigarette butts.

Some of the prepared samples are shown in Figure 2. As can be seen, the samples present a particular appearance, as they are nonhomogeneous. Indeed, three different porosities can be distinguished: Firstly, the porosity due to the presence of voids among the different butts and among butts and the impedance tube wall (we can call it external porosity); secondly, the porosity peculiar to the butts (voids between the fibers of the butts) (filter porosity); and, finally, the porosity that the fibers that compound the filter of the cigarette butt could have (fiber porosity). This triple porosity gives the samples a complicated structure.

### 2.3. Statistical Analysis

A variability analysis was performed based on the curves of the absorption coefficients and their Pearson’s coefficient of variation (CV). Descriptive statistics, such as the mean, standard deviation, and minimum and maximum values have been considered. Additionally, 95% confidence intervals for the mean value of the absorption coefficient are presented.

The coefficient of variation (CV) is defined as the relationship between the standard deviation of the sample and the average value. It measures the relative dispersion of the data (to what extent the data is close or far from its average). As this coefficient is dimensionless, it can be used to compare different datasets regardless of the units of measure.

An analysis of variance for the functional data (functional ANOVA) has been considered to report statistically significant differences between the curves of different groups of samples in the full frequency spectrum [37]. Data were transformed to 1/3 of the octave bands and one-way ANOVA and a *t*-test (with Bonferroni correction) have been applied to determine if there were statistically significant differences among the groups in those frequency bands.

Statistical analyses were performed using R software, release 3.6.0 [38]. Package fdANOVA [39] was used for the functional ANOVA. Two-sided *p* < 0.05 were considered statistically significant.

## 3. Results and Discussion

Firstly, a variability analysis is performed, then combinations of samples are compared and, finally, the one-third octave bands’ absorption coefficients were analyzed.

### 3.1. Variability Analysis

As can be seen in Table 1, several samples were prepared for each of the six groups. As mentioned, the cigarette butts used for the preparation of each group of samples have some similar characteristics (e.g., similar status, length, and diameter). Nevertheless, as the used butts can have a different precedence (brand, weather exposure, etc.) or a different compression exerted by user’s fingers, humidity, etc., it is important to study the observed variability among the different samples inside each combination.

**Figure 3:**
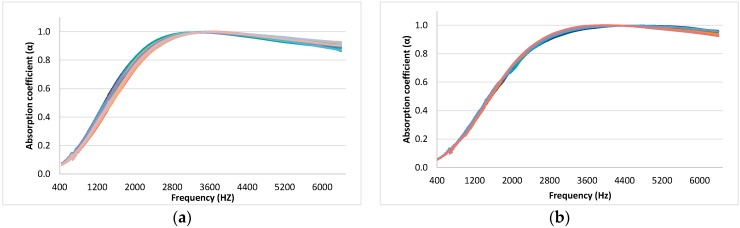

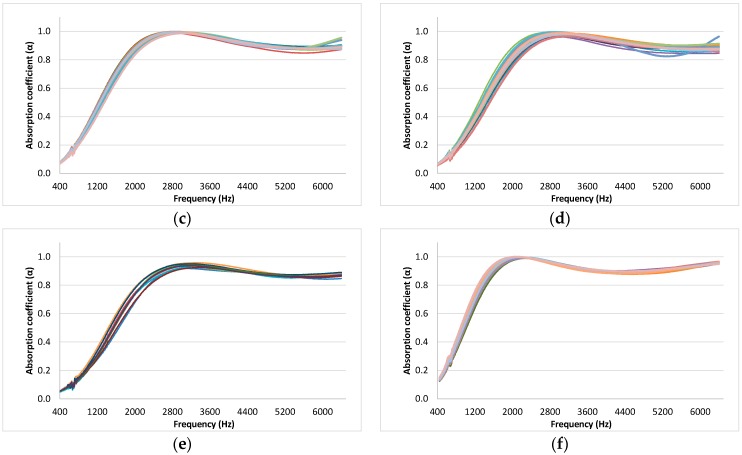
Absorption coefficient of the measured samples: (**a**) Samples from Group L1–C1; (**b**) Samples from Group L1–C4; (**c**) Samples from Group L2–C1; (**d**) Samples from Group L2–C2; (**e**) Samples from Group L2–C3; (**f**) Samples from Group L3–C1.

In a first step, all the absorption coefficients of the six different combinations of samples shown in Table 1 were represented (see Figure 3).

As can be seen in Figure 3, samples from each group do not present great variability. Despite the generally low variability, it was not the same for all the frequencies, as in some parts of the spectrum, the absorption coefficients of the different samples are more similar in value than in other parts. All measurements present a first maximum in the absorption coefficient, and the variability was higher under this absorption maximum. An increase in the variability can also be observed at higher frequencies (over 4500 Hz). Comparing the different groups, the variability of the graphs of samples with burnt butts (Figure 3d) and that of samples without paper (Figure 3e) was higher than the variability of the rest of the graphs.

**Figure 4:**
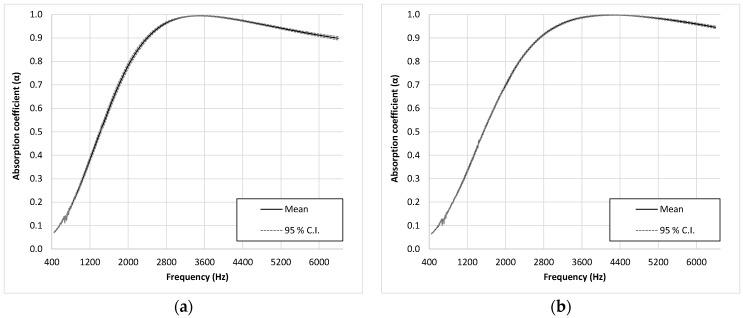

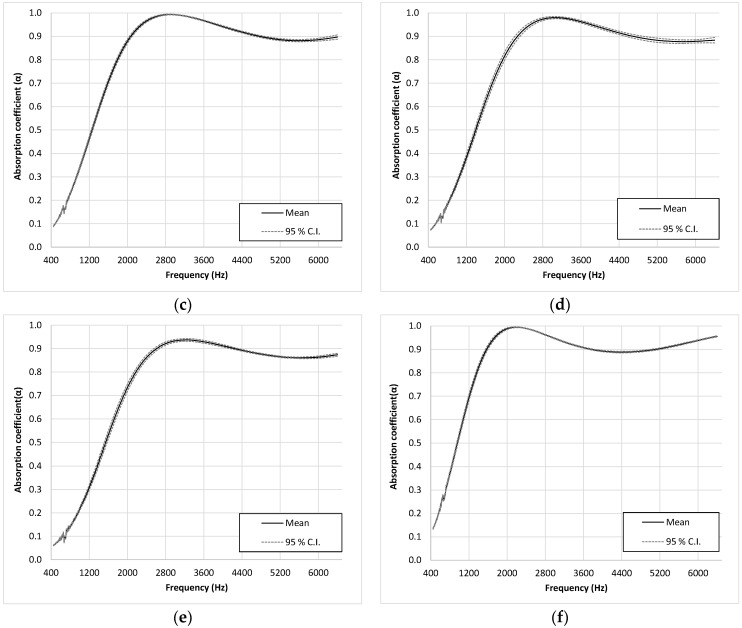
Mean value curves of the absorption coefficient, with 95% confidence interval curves: (**a**) Group L1–C1; (**b**) Group L1–C4; (**c**) Group L2–C1; (**d**) Group L2–C2; (**e**) Group L2–C3; (**f**) Group L3–C1.

The results shown in Figure 3 were then analyzed through an inferential analysis. For this purpose, 95% confidence intervals for the mean value of the absorption coefficient were calculated. The confidence intervals were very close to the mean, as shown in Figure 4. Therefore, these results confirm the low variability previously indicated in Figure 3.

**Figure 5:**
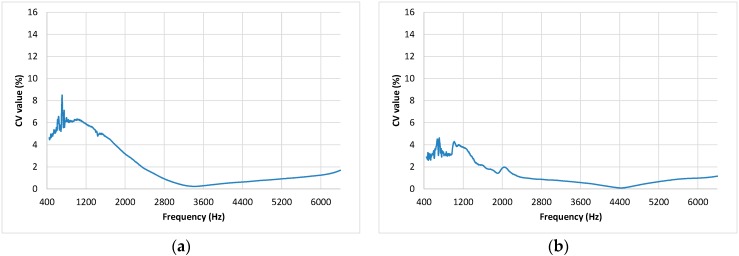

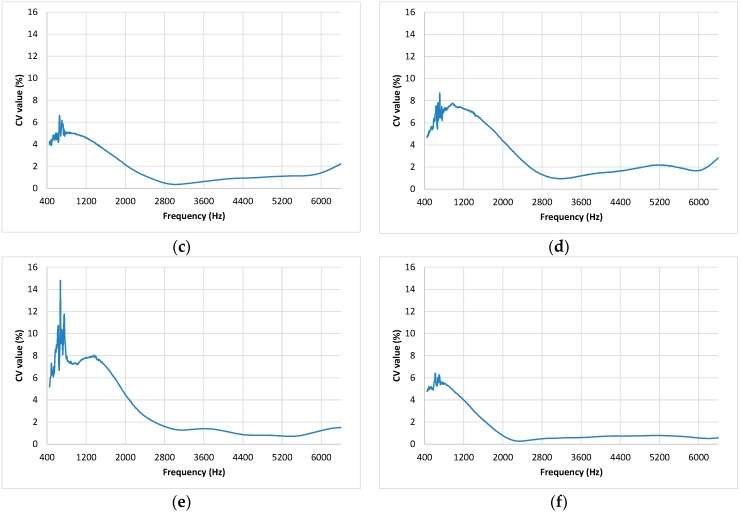
Coefficient of variation curves of the coefficient of absorption for the different groups of analyzed samples: (**a**) Group L1–C1; (**b**) Group L1–C4; (**c**) Group L2–C1; (**d**) Group L2–C2; (**e**) Group L2–C3; (**f**) Group L3–C1.

In addition to analyzing the absolute variability, the relative variability was evaluated using the coefficient of variation (CV). The CV obtained for the different frequencies is shown in Figure 5 and a descriptive analysis of the obtained values (mean, standard deviation, maximum, and minimum) is shown in Table 2.

As in the previous analyses, in Figure 5 and Table 2, it can be observed that all groups of samples have a low coefficient of variation. The coefficients of variability were less than 10% in all cases, except in some frequencies of group L2–C3, and in all cases, the averaged CV was lower than 3.03%. Comparing the different combinations of samples, the highest variability was found in the samples of groups L2–C2 and L2–C3. Group L2–C2 corresponds to burnt butts, and the fact that the size and position of the burnt zones were not equal in the different butts can explain this higher variability among the samples prepared with burnt cigarette butts. Group L2–C3 corresponds to the samples prepared with butts without the wrapping paper; when the paper is removed, the cigarette sometimes butt lost its shape, and this can be the reason for the increase in the variability among the samples of this group. With respect to frequencies, the range from 1000 to 2000 Hz is the most variable. At higher frequencies, a decrease in variability is first observed, then a slight new increase of variability is observed from 4400 Hz. Nevertheless, in general, the coefficient of variation is less than 2% from 2800 Hz onwards.

### 3.2. Comparison between Samples

In order to analyze whether the presence of burnt regions, the presence of wrapping paper, or the length in the butts of the samples have a significant influence on the absorption properties of the samples, we carried out a comparison of the groups of samples that could be considered similar in some way (located in the same row or in the same column in Table 1).

**Figure 6:**
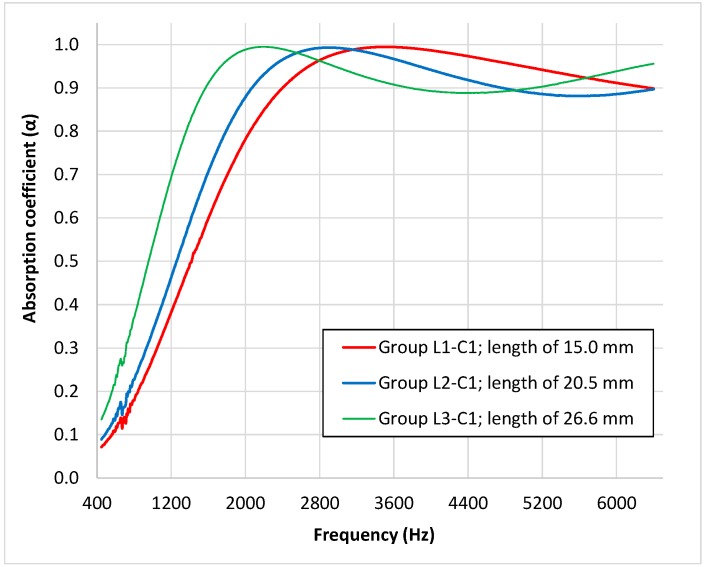
Mean value curves of the absorption coefficient for the three combinations of the samples of status 1.

To analyze the influence of the length of butts in relation to the acoustical behavior of the samples, the average absorption coefficient for the three combinations of the samples belonging to status 1 (the mean values of the absorption coefficient measured for these samples are shown in Figure 6) were compared.

**Figure 7:**
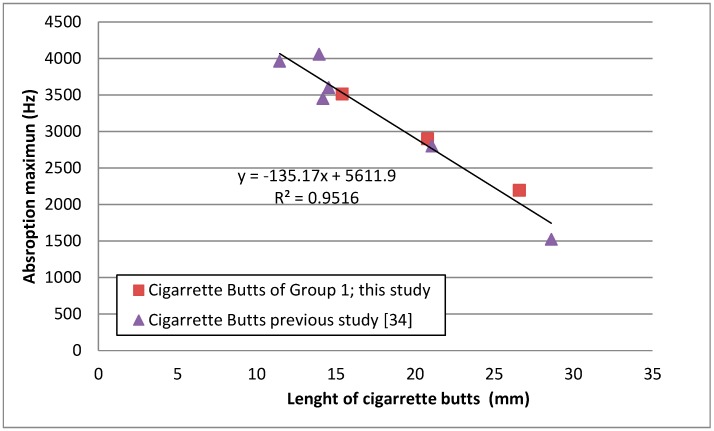
Variation of the frequency of maximum absorption with the length of cigarette butts of the samples.

It is known that the absorption of a porous material is due to the energy losses of the sound waves as moving air particles, induced by the wave, interact with the motionless particles near the skeleton or fibers of the porous material. Thus, the absorption is at its maximum when the maximum particle speed is located inside the absorbent material. As in the disposition of the measured samples, the porous material is faced with the rigid termination of the impedance tube, and near this rigid termination, there is a minimum particle speed with the maximum being located at a certain distance (λ/4) from this termination. Thus, when the thickness of the sample increases, the maximum absorption of the material will be shifted to lower frequencies. This shift was observed in the measured samples, as can be seen in Figure 6. Comparing the absorption coefficient curves, statistically significant differences were found (*p* < 0.001) using functional ANOVA for the three groups of samples. The shift in the maximum absorption frequency with the thickness of butt samples was studied in a previous work [34] showing that the relationship observed for butt samples was different to the observed for other sample (glass wool). The shift observed in the samples or the present research was congruent with the observed in a previous work [34] as can be observed in Figure 7, where results of both studies are compared.

**Figure 8:**
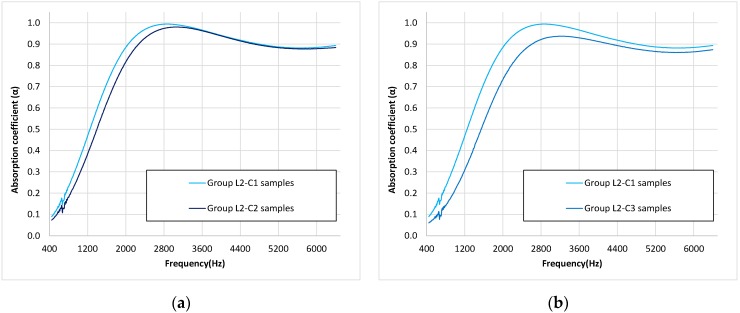
Mean value of the absorption coefficient for the three combinations of samples of lengths of around 20.5 mm. (**a**) Samples of used butts with paper and without burnt regions (Group L2–C1) are compared with samples of burnt butts (Group L2–C2); (**b**) Samples of used butts with paper and without burnt regions (Group L2–C1) are compared with samples composed of butts without paper and without burnt regions (Group L2–C3).

To analyze the influence of the presence of burnt regions and the effect of removing the wrapping paper on the absorption coefficient, the average absorption coefficient of these two combinations of samples (groups L2–C2 and L2–C3) were compared with the group of used butts with wrapped paper and without burnt regions (group L2–C1) (Figure 8). All these samples were composed of used butts with a similar length (about 20.5 mm).

As can be seen in Figure 8a, the maximum of absorption occurs at about the same frequency (around 3000 Hz) for both samples with and without burnt butts, and a similar absorption coefficient is observed for higher frequencies. Nevertheless, the absorption at frequencies under this maximum value were clearly lower than in the case of samples composed of used butts without burnt regions. The slight decrease in the absorption coefficient in the maximum value and the shift of this maximum to higher frequencies could be explained by considering that burnt regions represent a decrease in the number of fibers (they have collapsed in the significantly burnt regions) and, thus, the absorption efficiency of the material is reduced. This reduction of the absorption efficiency is not appreciable at higher frequencies, probably due to the fact that, at those frequencies, more than one maximum particle speed is inside the material. Despite the similar behavior at high frequencies, as mentioned, the functional ANOVA showed statistically significant differences (*p* < 0.001) between both groups (groups L2–C1 and L2–C2).

Regarding the effect of the wrapping paper, in Figure 8b, it can be seen that samples without this wrapping paper present both a lower absorption coefficient in all spectra, although the differences are lower with the frequency of the maximum absorption value. Additionally, a shift of the value of the frequency of the maximum of absorption to higher frequency values can be observed. The first effect (a decrease in the absorption efficiency) could be explained by the own absorption of the wrapping paper. It is important to note that the weight in the samples decreased by 34.1% ± 2.4% when the wrapping paper was removed. The second effect (a shift in the frequency of the maximum absorption to higher frequencies) would be consistent with the described decrease in the thickness of the sample, but the length of the butt of the samples was the same in both groups of samples. This shift can be explained, in this case, by the variation of the density of the absorber in the samples, which also produces a shift in the frequency of the maximum absorption, as previously described [34]. Due to important differences between both groups of samples, statistical differences were expected; thus, functional ANOVA also showed statistically significant differences (*p* < 0.001) between both sample groups (groups L2–C1 and L2–C3).

**Figure 9:**
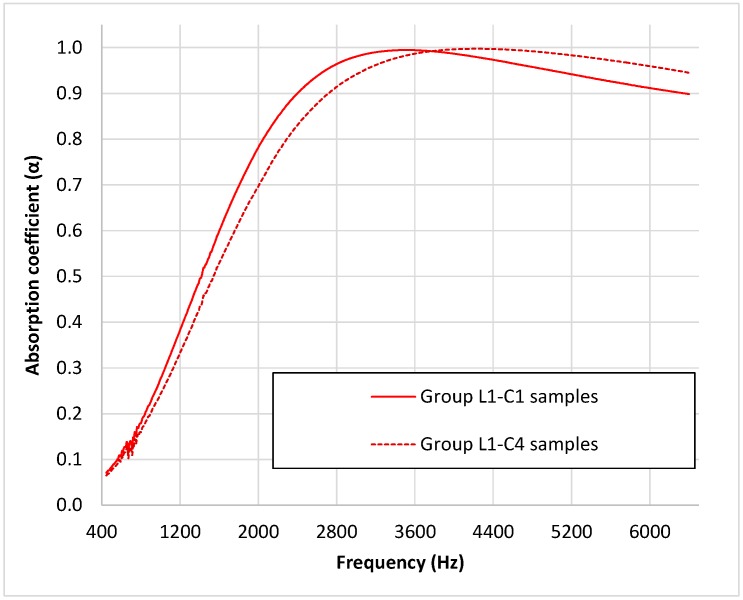
Mean value of the absorption coefficient for the two combinations of samples of a length of around 15.0 mm.

Finally, to evaluate the effect of smoking processes on the absorption properties of butts, samples composed of unsmoked butts (group L1–C4) were compared with samples composed of used butts with a similar length (group L1–C1) (both with butts of a length of approximately 15 mm) (see Figure 9). As can be seen in this figure, samples composed of unsmoked butts present a higher absorption with the frequency of the maximum absorption value than samples composed of used butts. Moreover, there is a shift of this maximum of absorption to higher frequencies in the case of unsmoked butt samples. As the unsmoked butt length is slightly lower than the length of the used ones with which they are being compared, the difference may also be explained by this difference in length, as shown in Figure 6. In this case, functional ANOVA also showed statistically significant differences (*p* < 0.001) between both sample groups (groups L1–C1 and L1–C4).

### 3.3. Analysis of the One-Third Octave Bands’ Absorption Coefficients

While functional ANOVA indicated that the overall absorption coefficients presented statistically significant differences among all the compared groups, in some frequency ranges of the graphs (i.e., for the high frequencies in Figure 8a), the observed curves were similar. For this reason, the initial coefficients of the absorption data were transformed into one-third octave bands, and then one-way ANOVA and a *t*-test (with Bonferroni correction) were used to compare the one-third octave means in the different groups of samples. The obtained *p*-values are shown in Table 3.

As can be seen, there is only one one-third octave band frequency in which differences were not statistically significant between Group L2–C1 (20.5 mm) and Group L1–C1 (15.0 mm) and, moreover, only one between Group L2–C1 (20.5 mm) and Group L3–C1 (26.6 mm). These two one-third octave band frequencies correspond to the overlap among the absorption coefficient data near the maximum value, which can be observed in Figure 6. With respect to Figure 7, the Group L2–C1 and Group L2–C2 results overlap at higher frequencies (4000, 5000, and 6400 Hz one-third octave bands) and, so, for these one-third octave band frequencies, no statistically significant differences were observed. For the rest of the pairwise comparisons, all the one-third octave bands presented statistically significant differences.

## 4. Conclusions

The studies, carried out with more than 100 samples, showed that samples prepared with used cigarette butts presented a high absorption coefficient. Thus, the obtained absorption coefficient values showed that the absorption capacity of the different measured samples were quite high, with absorption coefficients higher than 0.8 for frequencies over 2000 Hz.

According to this study, the absorption efficiency depends on the presence of burnt regions, the removal of the paper that wrapped the filter, and the length of the butts used for sample preparation. This effect was lower when the frequency was higher than the maximum of the absorption frequency.

Shifts in the frequency corresponding to the maximum value of the coefficient absorption were observed. These shifts could be explained by the different lengths of cigarette butts used in the sample preparation and the different densities of absorbing materials. This is consistent with the study presented in [34].

While some characteristics of the used butts could be different between samples (for instance, the humidity, degree of splashing, brand of the butts, etc.), the variability found in the measured samples can be considered low. Despite the low observed values of variability, it was observed that the presence of burnt regions in the used butt and the removal of the wrapped paper increased the variability of the samples.

## 5. Limitations of the Study and Further Studies

The results of this study, despite their potential, present some limitations. Firstly, the obtained absorption coefficients are valid only for perpendicular incidence. For real applications, a random incidence absorption coefficient would be desirable. Secondly, the absorption coefficients must be accompanied by other measured parameters such as flow resistivity, tortuosity, sample porosity, etc., in order to understand the acoustical behavior of samples.

With respect to the sample preparation, the chosen distribution of butts will probably not be adequate for a higher number of samples and other possibly more homogenous samples would be desirable.

To complete the analysis of the potential to use this recycled product in sound absorption applications in the construction industry, further studies are necessary which examine samples prepared with other configurations and samples prepared after cleaning the used butts prior to sampling preparation. Moreover, better knowledge of the properties of the samples (properties such as flow resistance, porosity, or tortuosity) would be advisable. Finally, comparisons with other conventional or sustainable materials would also be necessary.

## Figures and Tables

**Table 1:** Some properties (mean ± standard deviation) of the prepared samples. L = length; ϕ = diameter; *ρ* =density; por = porosity.

Length (L)	Cigarette Butts Status			
	C1 (Smoked without Burnt)	C2 (Smoked with Burnt)	C3 (Smoked without Burnt without Wrapping Paper)	C4 (Unsmoked with Wrapping Paper)
L1	20 samples L = 15.4 ± 0.1 mm ϕ = 8.01 ± 0.02 mm *ρ* = 140 ± 4 kg/m^3^ por = 0.929 ± 0.003	--	--	14 samples L = 14.7 ± 0.1 mm ϕ = 7.88 ± 0.01 mm *ρ* = 97 ± 2 kg/m^3^ por = 0.931 ± 0.001
L2	20 samples L = 20.8 ± 0.2 mm ϕ = 7.65 ± 0.07 mm *ρ* = 118 ± 7 kg/m^3^ por = 0.935 ± 0.008	20 samples L = 20.5 ± 0.2 mm ϕ = 7.63 ± 0.04 mm *ρ* = 123 ± 5 kg/m^3^ por = 0.936 ± 0.005	10 samples L = 20.9 ± 0.2 mm ϕ = 7.63 ± 0.08 mm *ρ* = 79 ± 3 kg/m^3^ por = 0.936 ± 0.010	--
L3	20 samples L = 26.6 ± 0.2 mm ϕ = 7.59 ± 0.04 mm *ρ* = 114 ± 2 kg/m^3^ por = 0.936 ± 0.005	--	--	--

**Table 2:** Average, standard deviation, and maximum and minimum of the CV values in a range from 504 to 6400 Hz for each group.

Group	Average	Standard Deviation	Minimum	Maximum
L1–C1	2.08	2.01	0.24	8.48
L1–C4	1.28	1.08	0.10	4.63
L2–C1	1.80	1.49	0.35	6.61
L2–C2	3.03	2.17	0.94	8.66
L2–C3	2.90	2.77	0.71	14.79
L3–C1	1.37	1.53	0.26	6.41

**Table 3:** Obtained *p*-values for the *t*-test, with Bonferroni correction, for the different comparisons. Differences that are not statistically significant are presented in bold.

	Obtained *p*-values					
1/3 Octave Band (Hz)	L1–C1 vs. L2–C1	L2–C1 vs. L3–C1	L1–C1 vs. L3–C1	L2–C1 vs. L2–C2	L2–C1 vs. L2–C3	L1–C1 vs. L1–C4
500	<0.001	<0.001	<0.001	<0.001	<0.001	<0.001
630	<0.001	<0.001	<0.001	<0.001	<0.001	<0.001
800	<0.001	<0.001	<0.001	<0.001	<0.001	<0.001
1000	<0.001	<0.001	<0.001	<0.001	<0.001	<0.001
1250	<0.001	<0.001	<0.001	<0.001	<0.001	<0.001
1600	<0.001	<0.001	<0.001	<0.001	<0.001	<0.001
2000	<0.001	<0.001	<0.001	<0.001	<0.001	<0.001
2500	<0.001	**0.26**	<0.001	<0.001	<0.001	<0.001
3150	**0.53**	<0.001	<0.001	<0.001	<0.001	<0.001
4000	<0.001	<0.001	<0.001	**0.30**	<0.001	<0.001
5000	<0.001	<0.01	<0.001	**0.49**	<0.001	<0.001
6400	<0.001	<0.001	<0.001	**0.08**	<0.001	<0.001
